# Classification of Blood Volume Decompensation State via Machine Learning Analysis of Multi-Modal Wearable-Compatible Physiological Signals

**DOI:** 10.3390/s22041336

**Published:** 2022-02-10

**Authors:** Yekanth Ram Chalumuri, Jacob P. Kimball, Azin Mousavi, Jonathan S. Zia, Christopher Rolfes, Jesse D. Parreira, Omer T. Inan, Jin-Oh Hahn

**Affiliations:** 1 Department of Mechanical Engineering, University of Maryland, College Park, MD 20742, USA; amousavi@umd.edu (A.M.); jessparr@umd.edu (J.D.P.); 2 Department of Electrical and Computer Engineering, Georgia Institute of Technology, Atlanta, GA 30308, USA; jacob.kimball@gatech.edu (J.P.K.); zia@gatech.edu (J.S.Z.); omer.inan@ece.gatech.edu (O.T.I.); 3 Global Center for Medical Innovation, Translational Training and Testing Laboratories, Inc. (T3 Labs), Atlanta, GA 30313, USA; Christopher.rolfes@devices.net

**Keywords:** hypovolemia, blood volume, machine learning, seismocardiogram, ballistocardiogram, wearables

## Abstract

This paper presents a novel computational algorithm to estimate blood volume decompensation state based on machine learning (ML) analysis of multi-modal wearable-compatible physiological signals. To the best of our knowledge, our algorithm may be the first of its kind which can not only discriminate normovolemia from hypovolemia but also classify hypovolemia into absolute hypovolemia and relative hypovolemia. We realized our blood volume classification algorithm by (i) extracting a multitude of features from multi-modal physiological signals including the electrocardiogram (ECG), the seismocardiogram (SCG), the ballistocardiogram (BCG), and the photoplethysmogram (PPG), (ii) constructing two ML classifiers using the features, one to classify normovolemia vs. hypovolemia and the other to classify hypovolemia into absolute hypovolemia and relative hypovolemia, and (iii) sequentially integrating the two to enable multi-class classification (normovolemia, absolute hypovolemia, and relative hypovolemia). We developed the blood volume decompensation state classification algorithm using the experimental data collected from six animals undergoing normovolemia, relative hypovolemia, and absolute hypovolemia challenges. Leave-one-subject-out analysis showed that our classification algorithm achieved an F1 score and accuracy of (i) 0.93 and 0.89 in classifying normovolemia vs. hypovolemia, (ii) 0.88 and 0.89 in classifying hypovolemia into absolute hypovolemia and relative hypovolemia, and (iii) 0.77 and 0.81 in classifying the overall blood volume decompensation state. The analysis of the features embedded in the ML classifiers indicated that many features are physiologically plausible, and that multi-modal SCG-BCG fusion may play an important role in achieving good blood volume classification efficacy. Our work may complement existing computational algorithms to estimate blood volume compensatory reserve as a potential decision-support tool to provide guidance on context-sensitive hypovolemia therapeutic strategy.

## 1. Introduction

Hypovolemia is a state of low blood volume and can be classified into absolute hypovolemia and relative hypovolemia [1]. Absolute hypovolemia is associated with the absolute deficit in circulating blood volume for normal blood vessel capacitance (e.g., hemorrhage, dehydration, and vomiting), while relative hypovolemia is associated with abnormally large vessel capacitance for normal blood volume (e.g., sepsis, vasoplegia, and heat stress). Hypovolemia is responsible for the majority of shock etiology and shock-induced mortality in the Emergency Department: in a study, absolute hypovolemia and relative hypovolemia combined accounted for 82% of shock (absolute hypovolemia 31% and relative hypovolemia 51%) and >73% of 7-day shock-induced mortality [2]. In general, absolute hypovolemia and relative hypovolemia are associated with distinct treatment strategies: absolute hypovolemia can be treated primarily by volume replenishment whereas relative hypovolemia is treated primarily by vasoactive drugs (although in practice both treatments may often be used together to maximize treatment efficacy). Hence, there is a clear need for early diagnosis of hypovolemia and its classification into absolute hypovolemia and relative hypovolemia in order to provide context-sensitive treatments.

Traditionally, vital signs such as blood pressure, urinary output, and heart rate have been employed as indicators of hypovolemia. To list a few, postural hypotension with the resulting dizziness and tachycardia were shown to be useful diagnostics of blood loss, whereas supine hypotension and tachycardia had no diagnostic value [3,4]. Urinary output is widely used as a therapeutic endpoint for diagnosing and resuscitating low blood volume states in burn injury patients [5]. However, overall sensitivity of these vital signs is low [3,4]. In addition, postural challenges may not be well suited to critically ill hypovolemia patients, which further weakens the utility of the vital sign-based hypovolemia indicators.

With the increasing body of work suggesting that vital signs alone are not effective in diagnosing hypovolemia [6], novel surrogate measures of hypovolemia have been investigated. These include serum lactate level and its clearance [4,7], dynamic indices such as systolic pressure variability and pulse pressure variability [8], and peripheral tissue perfusion parameters [9] to list a few. In some studies, vital signs and laboratory values were integrated into a hypovolemia screening tool [10,11,12]. More recently, notable advances in signal processing and machine learning (ML) have fostered the application of these techniques in hypovolemia diagnosis. Representative examples include discrete Fourier transform-based analysis of arterial blood pressure waveform [13], fast Fourier transform-based analysis of central venous blood pressure waveform [14], ML analysis of arterial blood pressure waveform in its entirety (known as “Compensatory Reserve Index”) [15], support vector machine analysis of compressed arterial blood pressure waveform via principal components analysis [16], ML analysis of photoplethysmogram (PPG) signal features derived from time-frequency analysis [17], deep learning analysis of electronic medical records, vital signs, and laboratory values [18,19], and natural language processing-aided voting ensemble ML analysis of pulse pressure and unstructured clinical notes [20].

Despite the long-standing effort, existing hypovolemia diagnosis measures and techniques have a few practical limitations. First, gold standard vital signs measurements (e.g., catheter-based arterial and venous blood pressure) and laboratory values for accurate computation of vital signs and dynamic indices are not always available, especially in austere environments and low-resource settings, presenting significant challenges in the continuous assessment of hypovolemia based on rudimentary vital signs and dynamic indices. Second, dynamic indices are effective only in mechanically ventilated patients but not in spontaneously breathing patients [21], which limits their applicability outside the intensive care settings. Third, prior effort to exploit multi-modal physiological signals in the context of hypovolemia diagnosis appears to be very much limited, despite the ongoing success in the development of various medical devices that can acquire multi-modal physiological signals [22,23]. Fourth, and perhaps most importantly, the vast majority of existing work has focused predominantly on the discrimination of either absolute hypovolemia (specifically, hemorrhage) or relative hypovolemia (specifically, sepsis) aspect of hypovolemia from normovolemia, while the capability for classifying hypovolemia into absolute hypovolemia and relative hypovolemia (which is important in making appropriate treatment decisions) has been largely neglected. Accordingly, the efficacy of existing methods in breaking hypovolemia down into absolute hypovolemia and relative hypovolemia is not known.

This paper intends to bridge the above gaps in hypovolemia diagnosis by (i) exploiting multi-modal wearable-compatible physiological signals to enable ubiquitous assessment of blood volume decompensation state even outside of intensive care settings and (ii) developing a novel computational algorithm to discriminate both absolute hypovolemia and relative hypovolemia from normovolemia. To the best of our knowledge, our algorithm is the first of its kind capable of discriminating normovolemia from hypovolemia as well as classifying hypovolemia into absolute hypovolemia and relative hypovolemia. In this way, it has the potential to help clinicians deliver context-sensitive treatments to hypovolemic patients. We realized our blood volume classification algorithm by (i) extracting a multitude of features indicative of blood volume decompensation state from multi-modal physiological signals including the electrocardiogram (ECG), the seismocardiogram (SCG), the ballistocardiogram (BCG), and the PPG, (ii) constructing two ML classifiers using the features, one based on random forest to classify normovolemia vs. hypovolemia and the other based on logistic regression to classify hypovolemia into absolute hypovolemia and relative hypovolemia, and (iii) sequentially integrating the two to enable multi-class classification (normovolemia, absolute hypovolemia, and relative hypovolemia). We developed and validated the blood volume decompensation state classification algorithm using the experimental data collected from six animals undergoing normovolemia, relative hypovolemia, and absolute hypovolemia challenges.

This paper is organized as follows. Section 2 presents a brief description of experimental data and details of the blood volume decompensation state classifier development. Section 3 presents results, which are discussed and interpreted in Section 4. Section 5 summarizes the paper with conclusions and lessons learned.

## 2. Materials and Methods

### 2.1. Experimental Data

**Figure 1:**
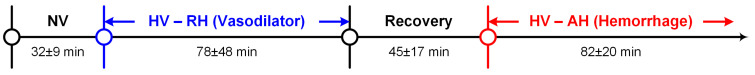
Experimental protocol consisting of a baseline (i.e., normovolemic (NV)) period followed by a relative hypovolemic (RH) period and an absolute hypovolemic (AH) period.

Experiments were performed in six Yorkshire swine (age: 114–150 days, weight: 52–71 kg) under the approval of the IACUC at the Georgia Institute of Technology (A100276), Translational Testing and Training Labs, Inc. (GT48P), and the Department of Navy Bureau of Medicine and Surgery. Full details of the experimental protocol are described in our prior work [24,25]. In brief, each animal was anesthetized and subject to a baseline period. Then, the animals underwent a relative hypovolemia period and then an absolute hypovolemia period (Figure 1). In relative hypovolemia, the animals received increasing doses of nitroglycerin until reaching one of two safety thresholds: (i) cardiovascular collapse defined as a 20% sustained drop in mean blood pressure or (ii) the maximum dosage of nitroglycerin, 500 mcg/min. In absolute hypovolemia, the animals were hemorrhaged through an arterial line at increments of 7% total blood volume (estimated by the Evans Blue dye technique) until reaching cardiovascular collapse. Each hemorrhage was paused for 5–10 min to allow the animals’ cardiovascular responses to stabilize. After the protocol, the animals were euthanized either by lethal injection of potassium chloride or exsanguination.

During the experiment, physiological signals including the ECG, the SCG, the BCG, and the PPG were measured at a 2 kHz sampling rate. These physiological signals can be measured simultaneously to yield features indicative of blood volume state (see Section 2.2 and Section 4.2 for details). The ECG was measured using electrodes placed in Lead II configuration and interfaced to a wired amplifier module (ECG100C, Biopac Systems, Goleta, CA, USA; ±10 V analog output with 0.1 µV rms noise in 0.5 Hz–35 Hz range). The SCG (anterior-posterior direction) and the BCG (superior-inferior direction) were measured using a 3-axis accelerometer (ADXL354, Analog Devices, Norwood, MA, USA; ±4 g range with 200 mV/g sensitivity, 20 µg/√Hz noise density, and 0.15 mg/°C temperature offset) placed on the mid-sternum and interfaced to a wired transducer interface module (HLT100C, Biopac Systems, Goleta, CA, USA). The PPG was measured using a trans-reflectance PPG sensor (TSD270A, Biopac Systems, Goleta, CA, USA) placed over a femoral artery and interfaced to a veterinary pulse oximeter module (OXY200, Biopac Systems, Goleta, CA, USA; 10 V analog output with 660 nm (red) and 910 nm (infrared) wavelengths). All the modules were interfaced with a data acquisition system (MP160, Biopac Systems, Goleta, CA, USA) with dedicated software (AcqKnowledge, Biopac Systems, Goleta, CA, USA). Note that all the physiological signals employed in this work are highly compatible with wearable sensing, as demonstrated in our prior work on patch [26] and wristwatch [27] devices.

### 2.2. Data Processing and Feature Extraction

The physiological signals were filtered using a finite impulse response zero-phase digital band-pass filter with a Kaiser window. The cut-off frequencies used were 0.5–40 Hz for the ECG, 1.0–40 Hz for the SCG and the BCG, and 0.5–10 Hz for the PPG, which were chosen based on the best practices reported in the literature [28,29]. Then, the R waves in the ECG were detected as local peaks. Subsequently, the signals were gated into individual cardiac beats using the ECG R wave as reference. Finally, the BCG was rotated to be perfectly perpendicular to the gravitational direction so that it maximally aligned with the superior-inferior direction.

**Figure 2:**
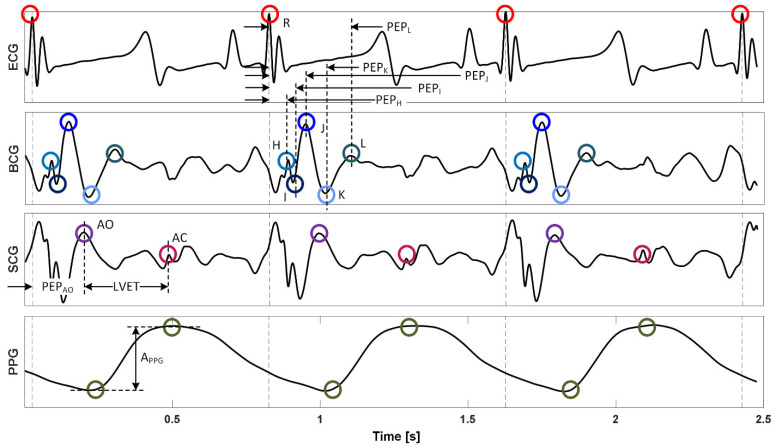
Representative physiological signal waveforms, fiducial points, and features. R: ECG R wave. H, I, J, K, and L: BCG H, I, J, K, and L waves. AO and AC: SCG AO and AC points.

A total of 46 features were extracted from the fiducial points derived from multiple physiological signals considered in this work [24,30] (Figure 2):

(1) Heart rate and heart rate variability based on three different methods [31] were calculated, including (i) time-domain method HRV_T_, (ii) Poincare method HRV_P_, and (iii) frequency-domain method HRV_F_. These constituted 4 features (HRV_T_, HRV_P_, HRV_F_, and heart rate).
(2) Cardiac timing intervals including pre-ejection period (PEP, as the time interval between the R wave in the ECG and (i) the AO [32] point in the SCG as well as (ii) the H, I, J, K, and L waves in the BCG; thus 6 in total) and left ventricular ejection time (LVET, as the time interval between the AO point and the AC point [32] in the SCG) as well as their ratios (PEP/LVET; 6 based on 6 PEPs) were calculated. These constituted 13 features.
(3) PPG amplitude (A_PPG_, as the vertical difference between the diastolic trough and systolic peak) was calculated as a measure of peripheral vasoconstriction/vasodilation. This constituted one feature.
(4) Various time intervals and amplitudes based on the fiducial points in the BCG were calculated: H, I, J, K, and L wave amplitudes, a total of 10 wave-to-wave time intervals and amplitudes (H-I, I-J, J-K, I-K, and K-L), and the variability associated with all these time intervals and amplitudes (as the standard deviation of the 100 causal beats preceding the cardiac beat of interest) were calculated (except the I-K interval and amplitude since I-K amplitude showed a very large coefficient of variation). These constituted 28 features.

All the features were normalized by their respective average values in the baseline state (i.e., normovolemia in Figure 1) on an individual basis. Then, all the normalized features were scaled using a standard scaler before developing and evaluating the blood volume decompensation state classifiers. Note that the scaler was determined solely based on the training dataset and then employed to transform the test dataset.

### 2.3. Classification of Blood Volume Decompensation State

**Figure 3:**
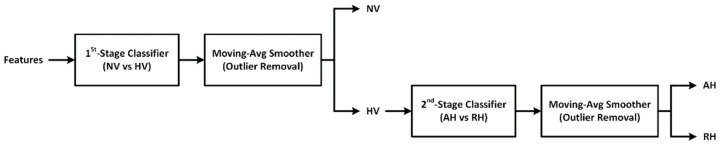
Machine learning (ML)-based multi-class blood volume decompensation state classification to discriminate normovolemia (NV) and hypovolemia (HV) as well as to classify hypovolemia into absolute hypovolemia (AH) and relative hypovolemia (RH).

To realize a multi-class classifier that can discriminate normovolemia and hypovolemia as well as classify hypovolemia into absolute hypovolemia and relative hypovolemia, two binary ML classifiers were developed based on the features extracted in Section 2.2 and then combined. The 1st-stage classifier determines if a subject is in normovolemia or hypovolemia state, while the 2nd-stage classifier determines if the subject is in absolute hypovolemia or relative hypovolemia in case the outcome of the 1st-stage classifier is hypovolemia (Figure 3). A post-processing step based on moving-average smoothing was employed in both stages to ensure robust classification by eliminating isolated misclassification instances (Figure 3). To make an efficient use of the limited data with a relatively small sample size (N = 6), we used the leave-one-subject-out cross-validation analysis in developing and validating the blood volume decompensation state classifiers.

#### 2.3.1. ML-Based Blood Volume Decompensation State Classifier: Development

Using the features described in Section 2.2, we performed preliminary feature selection using the wrapper method [33]. We selected optimal sets of features associated with the three ML classifiers considered in this work: (i) logistic regression, (ii) random forest, and (iii) support vector machine. In our experimental data, the number of hypovolemia samples was approximately 10 times larger than the number of normovolemia samples due to the much longer duration associated with hypovolemia than normovolemia (Figure 1), while hypovolemia consisted of absolute hypovolemia samples and relative hypovolemia samples at an approximate ratio of 3:1. To minimize the adverse impact of imbalance in classes on optimal feature selection, we used two remedies: (i) we applied resampled data to the wrapper method, and (ii) we used and optimized performance metric suited to the data characteristics associated with each classification stage in leave-one-subject-out cross-validation analysis, namely, F1 score for 1st -stage classifier and accuracy for 2nd-stage classifier. In selecting features to classify normovolemia and hypovolemia, we down-sampled hypovolemia samples because normovolemia samples from the baseline period were associated with stable and homogeneous physiological states (Figure 1) and over-sampling them may not provide rich data for feature selection. In selecting features to classify absolute hypovolemia and relative hypovolemia, we did not perform resampling because all the animals underwent heterogeneous physiological changes during both absolute hypovolemia and relative hypovolemia periods and the samples were just modestly imbalanced (Figure 1).

We optimized the features and hyper-parameters associated with each of the three ML classifiers sequentially as follows. First, we optimized the features for each ML classifier with an initial set of hyper-parameters using the wrapper method. In the case of the logistic regression classifier, we employed the L_2_ regularization to effectively minimize the requisite features for classification during its training. In the case of the random forest classifier, we built a random forest consisting of 100 estimators. We selected the maximum tree depth of 12 and the minimum requisite samples of 2 at the split nodes and 1 at the leaf nodes by minimizing the Gini impurity. During its training, we generated decision trees in the random forest by bagging and maximized the aggregated vote from multiple decision trees in the random forest. In the case of the support vector machine, we used a support vector machine with radial basis functions as kernels. During its training, we used the unity regularization parameter and the inverse of the product of the number of features and the variance of the training dataset as the kernel coefficient. Second, we optimized the hyper-parameters of the three ML classifiers, each equipped with its respective optimal features, using the random search hyper-parameter optimization method [34] via leave-one-subject-out cross-validation analysis.

The final multi-class classifier was realized by combining the best 1st-stage and 2nd-stage classifiers in cascade (Figure 3). The 1st-stage and 2nd-stage classifiers were followed by a 500-point moving average smoother and a 50-point moving average smoother, respectively, that filters the time series sequence of classification outcomes and removes isolated misclassification instances as outliers. For the 1st-stage classifier, normovolemia samples were labeled as “0” and hypovolemia samples were labeled as “1”. Hence, a sample input to the 1st-stage classifier was classified as normovolemia if the classifier output after smoothing was <0.5 and as hypovolemia otherwise. For the 2nd-stage classifier, relative hypovolemia samples were labeled as “0” and absolute hypovolemia samples were labeled as “1”. Hence, a sample inputted to the 2nd-stage classifier was classified as relative hypovolemia if the classifier output after smoothing was <0.5 and as absolute hypovolemia otherwise. Note that the classification threshold of 0.5 is a naïve choice and may be optimized to maximize the classifier performance, although such a refinement was not considered in this work given its primary interest in demonstrating the initial proof-of-concept of the proposed approach.

#### 2.3.2. ML-Based Blood Volume Decompensation State Classifier: Evaluation

The efficacy of the multi-class blood volume decompensation state classifier developed in Section 2.3.1 was evaluated using the leave-one-subject-out cross-validation analysis. For each animal, we developed a final multi-class classifier as described in Section 2.3.1 using the data pertaining to the remaining five animals. Then, the entire time series data of the animal was inputted to the final multi-class classifier on a beat-by-beat basis for classification into normovolemia vs. hypovolemia and then hypovolemia into absolute hypovolemia vs. relative hypovolemia. The classification efficacy was evaluated in terms of (i) accuracy, precision, recall, and F1 score pertaining to the 1st-stage classifier and the 2nd-stage classifier, respectively, and (ii) overall accuracy and F1 macro score pertaining to the multi-class classifier.

We had a particular interest in demonstrating (i) the superiority of our ML classifier to conventional vital sign-based techniques and (ii) the advantages associated with the use of multi-modal SCG-BCG fusion in blood volume decompensation state classification. Hence, we developed three multi-class ML classifiers for comparison purposes: (i) a classifier solely based on vital signs (heart rate, three heart rate variability measures, and blood pressure (systolic, diastolic, and mean)), (ii) a classifier incorporating the features based on the fiducial points in ECG, SCG, and PPG (but not BCG), and (iii) a classifier incorporating the features based on the fiducial points in ECG, BCG, and PPG (but not SCG). We developed and evaluated these competing classifiers exactly in the same way as our final multi-class classifier.

We performed feature importance analysis on our final multi-class classifier to interpret and gauge the relevance of the features selected to classify normovolemia vs. hypovolemia as well as to classify hypovolemia into absolute hypovolemia vs. relative hypovolemia. We in particular leveraged our prior work on a mathematical model-based analysis of the BCG [35,36,37] as well as work of others [38,39] to garner useful insights on the relevance of the selected features in classifying the blood volume decompensation state. 

## 3. Results

**Figure 4:**
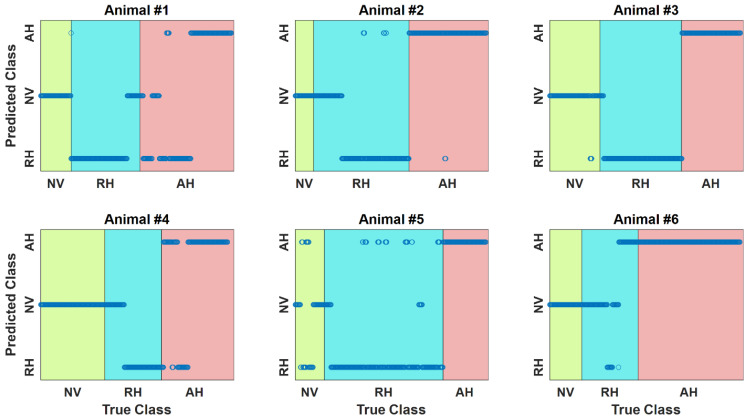
Time series sequences of multi-class blood volume decompensation state (normovolemia-relative hypovolemia-absolute hypovolemia) classification outcomes associated with all the animals in conjunction with the ground truth.

**Figure 5:**
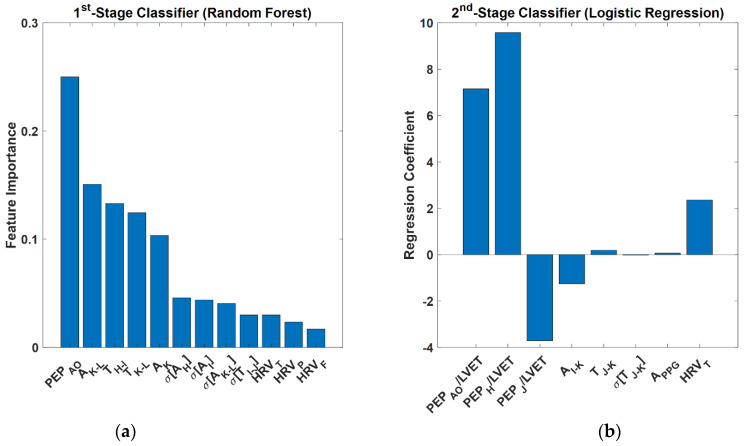
Feature importance associated with the best 1st-stage classifier (**a**) and best 2nd-stage classifier (**b**). Features are all normalized and thus unitless.

Table 1 and Table 2 present animal-by-animal and aggregated (in terms of mean and standard deviation) 1st-stage (normovolemia vs. hypovolemia) classification performance and 2nd-stage (hypovolemia into absolute hypovolemia vs. relative hypovolemia) classification performance associated with all the three candidate ML classifiers considered in this work (each pertaining to the optimal sets of features and hyper-parameters selected as described in Section 2.3.1). Table 3 shows animal-by-animal and aggregated (in terms of mean and standard deviation) multi-class classification performance associated with the best multi-class classifier (constructed by combining the best 1st-stage and 2nd-stage classifiers with a post-classification moving-average smoother in both stages as described in Section 2.3.1), while Table 4 shows the corresponding confusion matrix. Figure 4 shows the time series sequences of multi-class blood volume decompensation state classification outcomes associated with all the animals in conjunction with the ground truth. Table 5 compares (a) accuracy and (b) F1 macro scores associated with our final multi-class classifier and three competing ML classifiers (vital signs only, SCG-based features excluded, and BCG-based features excluded; see Section 2.3.2 for details). Figure 5 shows the feature importance associated with the best 1st-stage and 2nd-stage classifiers: Figure 5a shows the feature importance associated with the best 1st-stage classifier (i.e., random forest) in terms of minimum decrease in impurity, while Figure 5b shows the feature importance associated with the best 2nd-stage classifier (i.e., logistic regression) in terms of its coefficients.

## 4. Discussion

Detecting hypovolemia and classifying it into absolute hypovolemia and relative hypovolemia is important to enabling context-sensitive treatment of hypovolemia. However, the existing body of work has predominantly focused on the discrimination of either absolute hypovolemia or relative hypovolemia from normovolemia. The specificity of existing absolute hypovolemia detection methods against relative hypovolemia as well as the specificity of existing relative hypovolemia detection methods against absolute hypovolemia are not known. In addition, many existing methods rely on measurements that are not readily available in austere environments. Hence, there is a clear clinical need in enhancing the ability to classify hypovolemia into absolute hypovolemia and relative hypovolemia based on ubiquitously accessible measurements. This work intends to bridge these gaps, perhaps for the first time, by developing an ML-based hypovolemia classification algorithm based on multiple wearable-compatible physiological signals.

### 4.1. Blood Volume Decompensation State Classification Performance

The ML-based classifiers exhibited adequate performance in discriminating normovolemia vs. hypovolemia (Table 1) as well as in classifying hypovolemia into absolute hypovolemia and relative hypovolemia (Table 2). For 1st-stage classification, random forest exhibited the best performance in terms of both the mean and spread (i.e., standard deviation) associated with all the metrics (Table 1). For 2nd-stage classification, logistic regression exhibited the best performance in terms of both the mean and spread associated with accuracy (which is the metric optimized for 2nd-stage classification) (Table 2). Hence, we used random forest and logistic regression as 1st-stage and 2nd-stage classifiers, respectively. After conducting hyper-parameter optimization, the 2nd-stage logistic regression hyper-parameters did not change. In contrast, the 1st-stage random forest hyper-parameters changed as follows: (i) the number of estimators were chosen to be 672; (ii) the maximum tree depth decreased to 10; (iii) the minimum requisite samples at the split nodes increased to 10; and (iv) the minimum requisite samples at the leaf nodes increased to 2. It is worth noting that both 1st-stage and 2nd-stage classifiers were superior to naïve classifiers that always classify blood volume state to hypovolemia (accuracy: 0.89 vs. 0.80, F1 score: 0.93 vs. 0.89) and classify hypovolemia to absolute hypovolemia (accuracy: 0.89 vs. 0.48, F1 score: 0.88 vs. 0.64), respectively (note that the performance gap associated with 1st-stage classifier is small due to the large bias in the dataset). It is also worth noting that while the separation of normovolemia and hypovolemia required random forest-based nonlinear analysis of features, absolute hypovolemia and relative hypovolemia could be separated both by random forest and logistic regression, suggesting that a reasonably linear hyperplane that separates absolute hypovolemia and relative hypovolemia may be created using the wearable-compatible physiological signals. Remarkably, the features selected by logistic regression and random forest for classifying absolute hypovolemia and relative hypovolemia were quite similar (random forest selected PEP_H_/LVET, PEP_I_/LVET, and PEP_J_). The results are also consistent with the confusion matrix, which indicates that separating normovolemia and hypovolemia may be more challenging than separating absolute hypovolemia and relative hypovolemia (Table 4).

The multi-class classifier obtained by integrating the best 1st-stage and 2nd-stage classifiers along with a moving-average smoother at the end of both stages showed 81% accuracy and 77% F1 macro score on the average (Table 3 and Figure 4), which outperformed all the three competing ML classifiers (Table 5). In particular, our multi-class classifier was significantly superior to the one based only on the vital signs (average performance improved by 34% in accuracy and 36% in F1 macro score, *p* < 0.01; Table 5). In addition, it provided a large improvement in average performance relative to the classifier without the BCG-based features (accuracy by 12% and F1 macro score by 9%; Table 5) as well as a small improvement in average performance relative to the classifier without the SCG-based features (accuracy by 4% and F1 macro score by 2%; Table 5). A possible interpretation of these results is that (i) vital signs alone (even with invasive BP) are not sufficient to determine BV decompensation state; (ii) including only the SCG-based features as in our prior work [21,22] may not be not good enough to achieve good performance; and (iii) including both SCG-based and BCG-based features makes it possible to improve the overall performance. Hence, the results clearly demonstrate that ML analysis of multi-modal wearable-compatible physiological signals is a promising approach to continuous BV decompensation state classification, and that SCG-BCG fusion is advantageous in maximizing the classification efficacy.

Although rigorous analysis may not be feasible, we speculate that the results presented in this work are robust against metrological (especially noise) characteristics of the sensors used in this work. In particular, features most susceptible to measurement noise are the amplitudes (and time intervals to a lesser extent) derived from the BCG. Our calculation suggests that the average magnitudes of the BCG amplitude features are >100 times larger than the peak noise anticipated from the accelerometer. 

### 4.2. Feature Importance and Interpretation

Although complete interpretation and verification of the features selected to develop the ML-based blood volume decompensation state classifier was far from trivial, it appeared that many of the important selected features were relevant and adequate according to the existing physical knowledge and prior findings.

Features that played an important role in the 1st-stage classifier included: (i) PEP_AO_, (ii) BCG K-L and K wave amplitudes, (iii) BCG H-I and K-L wave time intervals, (iv) variability in the BCG H, I, and K-L wave amplitudes as well as I-J time interval, and (v) all the heart rate variability measures (Figure 5a). First, the relevance of PEP_AO_ may be supported by the physics of cardiac function, which suggests that PEP may increase with hypovolemia due to the reduction in the cardiac preload [40,41]. Second, the relevance of BCG K-L and K wave amplitudes may be supported by the physical mechanism underlying the genesis of the BCG [35,36]. More specifically, our prior mathematical model-based analysis shows that (i) the J and J-K waves in the conventional displacement BCG are approximately proportional to central aortic pulse pressure and distal aortic pulse pressure, respectively [35] and that (ii) the K and L waves in the acceleration BCG (i.e., the acceleration associated with body movement due to heartbeat, as the one measured in our work) approximately correspond to the J and K waves in the displacement BCG [36]. Assuming that central aortic pulse pressure is approximately proportional to cardiac SV and that distal aortic pulse pressure is in general proportional to its central aortic counterpart, the BCG K and K-L wave amplitudes are likely surrogates of cardiac SV. Hence, the BCG K and K-L wave amplitudes may manifest the influence of hypovolemia on preload. Third, our prior mathematical model-based analysis also shows that the BCG K-L wave interval exhibits inversely proportional relationship to pulse wave velocity [36], despite to a modest extent. Hence, it is speculated that hypovolemia is accompanied by low blood pressure, which in general decreases pulse wave velocity and increases the BCG K-L wave interval. On the other hand, the relationship between hypovolemia and the BCG H-I wave interval is not clear. Fourth, we do not know the exact physical mechanism between hypovolemia and the variability in the BCG features. However, our data analysis shows that the variability in the BCG features appear to increase during hypovolemia. Hence, the selection of the BCG feature variability as meaningful signatures of hypovolemia is not surprising, although deeper mechanistic understanding of how the variability in the BCG features is influenced by hypovolemia remains an open question. Fifth, the relevance of heart rate variability measures may be supported by the extensive body of existing literature reporting that (i) HRV_T_ and HRV_F_ are surrogates of sympathetic activity during hemorrhage and may be inversely related to central blood volume [42,43,44] (supporting its relationship to absolute hypovolemia), and that (ii) heart rate variability is correlated with the severity of systemic infection (e.g., sepsis) and may have diagnostic and prognostic value [45,46] (supporting its relationship to relative hypovolemia).

Features that played an important role in the 2nd-stage classifier included: (i) multiple PEP/LVET metrics (PEP_AO_/LVET, PEP_H_/LVET, and PEP_J_/LVET) and (ii) HRV_T_ (Figure 5b). On the one hand, the relevance of PEP/LVET metrics may be supported by the physics of cardiac function, which suggests that LVET may decrease with absolute hypovolemia due to the reduction in the cardiac stroke volume (SV) [40,41]. Hence, PEP/LVET increases as cardiac SV decreases, suggesting that an increase in PEP/LVET can be an indicator of absolute hypovolemia (in fact, we experimentally demonstrated the value of PEP/LVET in quantifying absolute hypovolemia in our prior work [24,25]). From this standpoint, the positive regression coefficients between absolute hypovolemia and PEP_AO_/LVET and PEP_H_/LVET are plausible. In contrast, the negative regression coefficient between absolute hypovolemia and PEP_J_/LVET is counterintuitive. We speculate that it may be the consequence of multi-collinearity, considering that PEP_AO_/LVET, PEP_H_/LVET, and PEP_J_/LVET in our data were highly correlated (PEP_AO_/LVET vs. PEP_H_/LVET: 0.82; PEP_AO_/LVET vs. PEP_I_/LVET: 0.75; PEP_H_/LVET vs. PEP_I_/LVET: 0.93). Not surprisingly, simply removing PEP_J_/LVET from the logistic regression classifier in the final classifier did not make any notable impact on the classification performance (accuracy: 0.78 +/− 0.11; F1 macro score: 0.74 +/− 0.12). On the other hand, the relevance of HRV_T_ may require further investigation. A prior work observed that HRV_T_ increases in absolute hypovolemia [44]. Yet, to our knowledge, there is no prior work investigating how HRV_T_ is influenced by relative hypovolemia. Our data analysis shows that HRV_T_ lacks consistency in terms of its changes due to absolute hypovolemia and relative hypovolemia. Hence, the role of HRV_T_ in separating absolute hypovolemia and relative hypovolemia must be scrutinized in follow-up work. 

### 4.3. Study Limitations and Future Opportunities

This study has a few limitations and implications for future opportunities. First, the sample size was small (N = 6). Hence, despite the promising results obtained in this paper, future work is needed to ascertain the efficacy of the blood volume decompensation state classifier and the robustness of the selected features in larger datasets. Second, the computations for blood volume decompensation state classification were performed offline using reasonably high-quality data obtained from anesthetized animals associated with minimal motion artifacts and sensor noises. To enable its real-world use, online signal processing methods for assessment of physiological signal quality, elimination of motion artifacts and sensor noises (including the variability originating from body postures [47] and measurement instruments [48]), robust detection of fiducial points in the physiological signals, and computation of features must be developed. Third, more extensive effort must be invested before the promise demonstrated in healthy animals in this work can be translated to humans who are possibly associated with diseases and comorbidities.

## 5. Conclusions

This paper presented a novel ML-based blood volume decompensation state classification algorithm that has the potential for implementation using ultra-convenient wearable devices. We demonstrated that multi-modal physiological signals can be analyzed using ML to yield adequate classification of blood volume decompensation state, that SCG and BCG signals can play meaningful and complementary roles in blood volume decompensation state classification, and that multi-modal physiological signal analysis has clear advantages over conventional vital signs-based techniques to blood volume decompensation state classification. Future work must conduct an extensive evaluation of the blood volume decompensation state classification algorithm in larger datasets as well as research on its real-time implementation.

## Figures and Tables

**Table 1:** Performance of 1st-stage classifier based on leave-one-subject-out analysis. Aggregated performance is shown in terms of mean and standard deviation.

**(a) Logistic Regression**				
**Animal**	**Accuracy**	**Precision**	**Recall**	**F1 Score**
1	0.77	0.94	0.78	0.85
2	0.93	0.93	1.00	0.96
3	0.64	1.00	0.52	0.68
4	0.74	0.71	1.00	0.83
5	0.79	0.97	0.77	0.86
6	0.83	0.97	0.82	0.89
Aggregated	0.78 ± 0.09	0.92 ± 0.10	0.81 ± 0.16	0.85 ± 0.08
**(b) Random Forest**				
**Animal**	**Accuracy**	**Precision**	**Recall**	**F1 Score**
1	0.88	1.00	0.86	0.92
2	0.85	1.00	0.84	0.91
3	0.97	0.99	0.97	0.98
4	0.93	1.00	0.89	0.94
5	0.89	0.92	0.96	0.94
6	0.84	1.00	0.81	0.89
Aggregated	0.89 ± 0.04	0.98 ± 0.03	0.89 ± 0.06	0.93 ± 0.03
**(c) Support Vector Machine**				
**Animal**	**Accuracy**	**Precision**	**Recall**	**F1 Score**
1	0.80	1.00	0.76	0.86
2	0.88	1.00	0.86	0.93
3	0.78	0.86	0.83	0.85
4	0.86	1.00	0.79	0.88
5	0.96	0.98	0.97	0.97
6	0.73	1.00	0.67	0.80
Aggregated	0.83 ± 0.07	0.97 ± 0.05	0.81 ± 0.09	0.88 ± 0.05

**Table 2:** Performance of 2nd-stage classifier based on leave-one-subject-out analysis. Aggregated performance is shown in terms of mean and standard deviation.

**(a) Logistic Regression**				
**Animal**	**Accuracy**	**Precision**	**Recall**	**F1 Score**
1	0.69	1.00	0.47	0.64
2	0.98	0.96	0.99	0.97
3	0.99	1.00	0.99	0.99
4	0.89	1.00	0.80	0.89
5	0.93	0.79	0.99	0.88
6	0.86	0.82	1.00	0.90
Aggregated	0.89 ± 0.10	0.93 ± 0.09	0.87 ± 0.19	0.88 ± 0.11
**(b) Random Forest**				
**Animal**	**Accuracy**	**Precision**	**Recall**	**F1 Score**
1	0.52	0.55	0.89	0.68
2	0.98	0.99	0.96	0.98
3	0.98	0.95	0.99	0.97
4	0.95	1.00	0.92	0.96
5	0.99	1.00	0.99	0.99
6	0.85	0.93	0.84	0.88
Aggregated	0.88 ± 0.16	0.90 ± 0.16	0.93 ± 0.05	0.91 ± 0.10
**(c) Support Vector Machine**				
**Animal**	**Accuracy**	**Precision**	**Recall**	**F1 Score**
1	0.49	0.53	0.83	0.66
2	0.99	0.97	1.00	0.99
3	0.92	0.85	0.99	0.91
4	0.86	1.00	0.75	0.85
5	0.99	1.00	0.99	0.99
6	0.77	0.97	0.66	0.78
Aggregated	0.84 ± 0.17	0.89 ± 0.17	0.87 ± 0.13	0.86 ± 0.11

**Table 3:** Performance of multi-class classifier based on leave-one-subject-out analysis (1st-stage random forest and 2nd-stage logistic regression). Aggregated performance is shown in terms of mean and standard deviation. NV: normovolemia. HV: hypovolemia. AH: absolute hypovolemia. RH: relative hypovolemia.

Animal	Accuracy	Precision	Recall	F1 Macro Score
1	0.68	0.70	0.77	0.68
2	0.83	0.78	0.88	0.78
3	0.97	0.96	0.97	0.97
4	0.82	0.85	0.84	0.81
5	0.83	0.79	0.76	0.77
6	0.73	0.58	0.66	0.59
Aggregated	0.81 ± 0.09	0.78 ± 0.11	0.81 ± 0.09	0.77 ± 0.11

**Table 4:** Confusion matrix associated with the multi-class classifier (1st-stage random forest and 2nd-stage logistic regression). Green cells indicate correct classification. Pink cells indicate incorrect classification.

	NV (ML)	HV-RH (ML)	HV-AH (ML)
NV	13,205 (15.8%)	913 (1.0%)	385 (0.4%)
HV-RH	7595 (9.1%)	27,319 (32.6%)	3965 (4.7%)
HV-AH	950 (1.1%)	2345 (2.8%)	27,005 (32.2%)

**Table 5:** Comparison of final multi-class classifier and three competing ML classifiers (vital signs only, SCG-based features excluded, and BCG-based features excluded).

	Final Classifier	No SCG	No BCG	Vital Signs
Accuracy	0.81 ± 0.09	0.77 ± 0.08	0.69 ± 0.21	0.47 ± 0.12 ^†^
F1 Macro	0.77 ± 0.11	0.75 ± 0.09	0.68 ± 0.21	0.41 ± 0.17 ^†^

^†^: *p* < 0.01 (paired *t*-test).

## Data Availability

Data used to evaluate the conclusions in the paper are present in the paper. Additional data related to this paper may be requested from the authors. The data collected and analyzed in the animal study is publicly available on IEEE Dataport at https://ieee-dataport.org/open-access/wearable-and-catheter-based-cardiovascular-signals-during-progressive-exsanguination.
